# Tailoring second-line or above therapy for patients with advanced or metastatic gastric cancer: A multicenter real-world study

**DOI:** 10.3389/fphar.2022.1043217

**Published:** 2022-11-17

**Authors:** Caiyun Nie, Weifeng Xu, Huifang Lv, Xiaohui Gao, Guofeng Li, Beibei Chen, Jianzheng Wang, Yingjun Liu, Jing Zhao, Yunduan He, Saiqi Wang, Xiaobing Chen

**Affiliations:** ^1^ Department of Medical Oncology, Affiliated Cancer Hospital of Zhengzhou University, Henan Cancer Hospital, Zhengzhou, China; ^2^ State Key Laboratory of Esophageal Cancer Prevention and Treatment, Zhengzhou University, Zhengzhou, China; ^3^ Henan Engineering Research Center of Precision Therapy of Gastrointestinal Cancer, Zhengzhou, China; ^4^ Zhengzhou Key Laboratory for Precision Therapy of Gastrointestinal Cancer, Zhengzhou, China; ^5^ Department of Oncology, First Affiliated Hospital of Henan University of Science and Technology, Luoyang, China; ^6^ Department of Oncology, The First Affiliated Hospital of Henan University of CM, Zhengzhou, China; ^7^ Department of General Surgery, Affiliated Cancer Hospital of Zhengzhou University, Henan Cancer Hospital, Zhengzhou, China

**Keywords:** gastric cancer, chemotherapy, anti-angiogenesis, immunotherapy, tyrosine kinase inhibitors

## Abstract

**Background:** There is currently still a lack of effective therapeutic manner after the failure of first-line therapy for patients with advanced or metastatic gastric cancer. The present study aimed to evaluate the clinical efficacy and safety of different treatment strategies as second-line or above therapy for patients with advanced or metastatic gastric cancer.

**Methods:** This was an observational multicenter real-world study. From January 2018 to December 2020, advanced or metastatic gastric cancer patients who have failed prior therapy were enrolled and treated with chemotherapy, anti-angiogenic TKIs (tyrosine kinase inhibitors) + chemotherapy or TKIs + ICIs (immune checkpoint inhibitors). In this study, progression free survival (PFS) was the primary end-point. Other evaluation indicators were objective response rate (ORR), disease control rate (DCR), overall survival (OS) and drug toxicities.

**Results:** 162 patients were enrolled, of which 61 patients received chemotherapy, 47 patients received TKIs plus chemotherapy, and 54 patients received TKIs + ICIs. No statistically significant difference existed in ORR among groups (16.4% vs. 19.1% vs. 18.5%, *p* = 0.924). Patients who received TKIs plus chemotherapy obtained better DCR compared with the chemotherapy group (78.7% vs. 54.1%, *p* = 0.008), and simultaneously, the median PFS (3.3 m vs. 2.8 m, *p* = 0.001) and OS (8.0 m vs. 5.8 m, *p* = 0.005) in TKIs plus chemotherapy group were superior to chemotherapy group. Consistent results were observed in subgroup analysis, including sex, age, ECOG, number of metastatic sites and treatment line. No statistically differences were found between TKIs + ICIs and the chemotherapy group concerning DCR (63.0% vs. 54.1%, *p* = 0.336), median PFS (3.0 m vs. 2.8 m, *p* = 0.051) and OS (5.2 m vs. 5.8 m, *p* = 0.260). Different treatment manner present a special spectrum of adverse events (AEs), and the incidence of Grade 3–4 AEs were 31.1%, 38.3% and 18.5%, respectively.

**Conclusion:** Compared with chemotherapy, anti-angiogenic TKIs plus chemotherapy demonstrated superior second-line or above therapeutic efficacy for advanced or metastatic gastric cancer with well tolerated toxicity. However, TKIs + ICIs failed to demonstrate a clinical advantage over chemotherapy.

## Introduction

Cancer epidemiological data showed that the incidence of gastric cancer is increasing year by year in the world (Sung et al., 2021). With the deepening of understanding of the pathogenesis and molecular changes of gastric cancer, as well as the progress of new comprehensive treatment methods, the overall treatment level of gastric cancer and the prognosis of patients has been significantly improved in recent years (Korfer et al., 2021; Hogner and Moehler, 2022). However, a considerable proportion of patients with gastric cancer are in the advanced or metastatic stage at the time of initial diagnosis. There is still no effective treatment option for advance or metastatic gastric cancer, which is a bottleneck restricting the long-term survival of gastric cancer patient (Hackshaw et al., 2020; Parisi et al., 2020).

Currently, platinum- and fluorouracil-based chemotherapy is the recommended first-line treatment regimen for advanced or metastatic gastric cancer (Koizumi et al., 2008; Luo et al., 2010; Lu et al., 2018). The main scheme of the second-line treatment is single drug chemotherapy with paclitaxel and irinotecan or a combination of two drug chemotherapy schemes (Hawkes et al., 2011; Hironaka et al., 2013; Shitara et al., 2017). However, the therapeutic effect of gastric cancer second-line treatment is not satisfactory, and the median overall survival (OS) is less than 1 year (Hironaka et al., 2013).

Anti-angiogenic therapy and immunotherapy provide new treatment options for patients with advanced or metastatic gastric cancer. Based on the results of the RAINBOW, ATTRACTION-02 and KEYNOTE-059 clinical trial, anti-angiogenic ramucirumab combined with chemotherapy and immunotherapy were approved by the FDA for the second-line and third-line treatment of metastatic gastric cancer (Wilke et al., 2014; Kang et al., 2017; Fuchs et al., 2018), respectively.

Some small-sample retrospective studies suggested that anti-angiogenic small-molecule multi-targeted tyrosine kinase inhibitors (TKIs) combined with chemotherapy, immune checkpoint inhibitors (ICIs) plus anti-angiogenesis may be potentially effective combination therapy strategies (Gao et al., 2022; Li et al., 2022; Tang et al., 2022). The present study aimed to evaluate the clinical efficacy and safety of chemotherapy, anti-angiogenic TKIs + chemotherapy and TKIs + ICIs as second-line or above therapy options for advanced or metastatic gastric cancer.

## Materials and methods

### Patients

From January 2018 to December 2020, patients with advanced or metastatic gastric cancer who have failed prior treatment in 3 institutions across China were enrolled in this study, including the Affiliated Cancer Hospital of Zhengzhou University, First Affiliated Hospital of Henan University of Science and Technology and First Affiliated Hospital of Henan University of CM. The criteria included: 1) advanced and metastatic gastric cancer; 2) failed from prior treatment; 3) based on RECIST v1.1, the presence of at least one measurable lesion.

### Study design and treatment

In this observational multicenter real-world study, the patients received chemotherapy, anti-angiogenic TKIs + chemotherapy or TKIs + ICIs therapy as second-line and beyond until progressive disease, death or intolerable toxicity occurs. In the chemotherapy regimen group, the patients received irinotecan or paclitaxel monotherapy. Irinotecan was administered intravenously at a dose of 125 mg/m^2^ on d1 and d8 every 3 weeks. The paclitaxel was given intravenously, the dosage was 80 mg/m^2^ on d1, d8 and d 15 every 4 weeks. In the TKIs + chemotherapy regimen group, the patients received apatinib or anlotinib plus chemotherapy. Apatinib was administered orally, the dosage was 250 mg daily and anlotinib was administered at a dose of 10 mg once a day from d1 to d14 for 3 weeks. The chemotherapy was given at the recommended dose, including irinotecan or paclitaxel. In the TKIs + ICIs therapy regimen, apatinib or anlotinib was given at the same dose as the TKIs + chemotherapy regimen group and combined with ICIs. Concering ICIs, programmed cell death protein 1 (PD-1) inhibitor was administered intravenously at the recommended dose, including sintilimab, camrelizumab or tislelizumab.

### Efficacy and safety assessments

After every two cycles of treatment, imaging examinations were performed with CT or MRI. The clinical response was evaluated as complete response (CR), partial response (PR), stable disease (SD), and progressive disease (PD) according to RECIST version 1.1 response evaluation criteria in solid tumors. The ratio of CR plus PR was objective response rate (ORR), and the ratio of CR, PR, plus SD was disease control rate (DCR). The drug toxicities were evaluated using the Common Terminology Criteria for Adverse Events (version 4.0).

### Statistical analysis

Differences between groups were compared by Pearson’s chi-squared test or Fisher’s exact test. The follow-up period ends on 31 December 2021. Progression free survival (PFS) was calculated from using chemotherapy, TKIs + chemotherapy or TKIs + ICIs therapy to disease progression or death. Overall survival (OS) was calculated from using chemotherapy, TKIs + chemotherapy or TKIs + ICIs therapy to the death of the patient or end of follow-up. Kaplan-Meier method and the log-rank test were used to perform survival and prognostic analysis. Subgroup analysis of impact indicators for PFS was carried out by Cox proportional hazards model, including sex, age, ECOG, number of metastatic sites and treatment line. SPSS 22.0 software (SPSS Inc., IL, US) was used for statistical analysis, and *p* < 0.05 was defined as statistically significant.

## Results

### Patient and treatment

A total of 162 patients were included. Table 1 summarized the baseline clinicopathological and treatment characteristics of patients. The median age of patients in this study was 55 years (range 25–80), with 117 (72.2%) male patients and 45 (27.8%) female patients. ECOG PS was 0–1 in 115 (71.0%) patients and the other 47 (29.0%) patients were ECOG PS 2. The tumor site in 69.1% patients was gastric, and the other 30.9% patients were gastroesophageal junction (GEJ) adenocarcinoma. The common metastatic sites included lymph node (66.0%), liver (44.4%), peritoneum (28.4%), lung (21.0%) and other sites (35.2%). 113 (69.8%) patients had 1 or 2 metastatic sites, and the metastatic sites in other 49 (30.2%) patients were 3 or more. All the 162 patients had undergone previous systematic therapy, 66 (40.7%) received chemotherapy, TKIs + chemotherapy or TKIs + ICIs therapy as second-line therapy, and as third-fourth line therapy in the other 96 (59.3%) patients. In this study, patients received the guideline-recommended first-line treatment regimen. For HER2-negative patients, first-line chemotherapy is based on fluorouracil in combination with platinum and/or taxane. For HER2-positive patients, first-line therapy is chemotherapy combined with trastuzumab targeted therapy.

**TABLE 1:** Patient and treatment characteristics.

Characteristic	All patients n = 162 n (%)	Chemotherapy n = 61 n (%)	TKIs + chemotherapy n = 47 n (%)	TKIs + ICIs n = 54 n (%)	*P*
Age					0.280
Median (range)	55 (25–80)	53 (25–76)	55 (26–74)	59 (30–80)	
≥60	67 (41.4)	22 (36.1)	18 (38.3)	27 (50.0)	
<60	95 (58.6)	39 (63.9)	29 (61.7)	27 (50.0)	
Sex					0.178
Female	45 (27.8)	22 (36.1)	10 (21.3)	13 (24.1)	
Male	117 (72.2)	39 (63.9)	37 (78.7)	41 (75.9)	
ECOG					0.873
0–1	115 (71.0)	44 (72.1)	32 (68.1)	39 (72.2)	
2	47 (29.0)	17 (27.9)	15 (31.9)	15 (27.8)	
Primary tumor site					0.229
Gastric	112 (69.1)	39 (63.9)	37 (78.7)	36 (66.7)	
GEJ	50 (30.9)	22 (36.1)	10 (21.3)	18 (33.3)	
Metastatic site					0.777
Lymph node	107 (66.0)	38 (62.3)	33 (70.2)	36 (66.7)	
Liver	72 (44.4)	31 (50.8)	22 (46.8)	19 (35.2)	
Peritoneum	46 (28.4)	15 (24.6)	14 (29.8)	17 (31.5)	
Lung	34 (21.0)	14 (23.0)	9 (19.1)	11 (20.4)	
Others	57 (35.2)	27 (44.3)	12 (25.5)	18 (33.3)	
Number of metastatic sites					0.841
1–2	113 (69.8)	41 (67.2)	33 (70.2)	39 (72.2)	
≥3	49 (30.2)	20 (32.8)	14 (29.8)	15 (27.8)	
Prior surgery					0.060
Yes	48 (29.6)	12 (19.7)	19 (40.4)	17 (31.5)	
No	114 (70.4)	49 (80.3)	28 (59.6)	37 (68.5)	
HER-2 status					0.944
Positive	22 (13.6)	9 (14.8)	6 (12.8)	7 (13.0)	
Negative	140 (86.4)	52 (85.2)	41 (87.2)	47 (87.0)	
Treatment line					0.919
2	66 (40.7)	26 (42.6)	19 (40.4)	21 (38.9)	
3–4	96 (59.3)	35 (57.4)	28 (59.6)	33 (61.1)	

Abbreviations: ECOG, eastern cooperative oncology group performance status; GEJ, gastroesophageal junction tumors; TKI, tyrosine kinase inhibitors; ICIs, immune checkpoint inhibitors.

Sixty-one patients received chemotherapy, of which paclitaxel and irinotecan were given in 29 (47.5%) and 32 (52.5%) patients, respectively. Forty-seven patients received anti-angiogenic TKIs combined with chemotherapy. In this group, 26 (55.3%) patients received apatinib plus irinotecan therapy, 14 (29.8%) patients received anlotinib plus paclitaxel therapy and the other 7 (14.9%) patients received anlotinib plus irinotecan regimen (Table 2). Fifty-four patients received anti-angiogenic TKIs combined with ICIs. Apatinib plus sintilimab was given in 25 (46.3%) patients, the other 29 (53.7%) patients received anlotinib plus ICIs, including sintilimab (11, 20.4%), camrelizumab (10, 18.5%) and tislelizumab (8, 14.8%). No statistically significant differences existed in the baseline clinicopathological characteristics among chemotherapy, TKIs + chemotherapy and TKIs + ICIs groups.

**TABLE 2:** Regimens given in chemotherapy, TKIs + chemotherapy and TKIs + ICIs for patients with advanced or metastatic gastric cancer.

Regimen	Number of patients (%)
Chemotherapy (n = 61)	
Paclitaxel	29 (47.5)
Irinotecan	32 (52.5)
TKIs + chemotherapy(n = 47)	
Apatinib + Irinotecan	26 (55.3)
Anlotinib + Paclitaxel	14 (29.8)
Anlotinib + Irinotecan	7 (14.9)
TKIs + ICIs(n = 54)	
Apatinib + Sintilimab	25 (46.3)
Anlotinib + Sintilimab	11 (20.4)
Anlotinib + Camrelizumab	10 (18.5)
Anlotinib + Tislelizumab	8 (14.8)

Abbreviations: TKI, tyrosine kinase inhibitors; ICIs, immune checkpoint inhibitors.

### Efficacy

**FIGURE 1:**
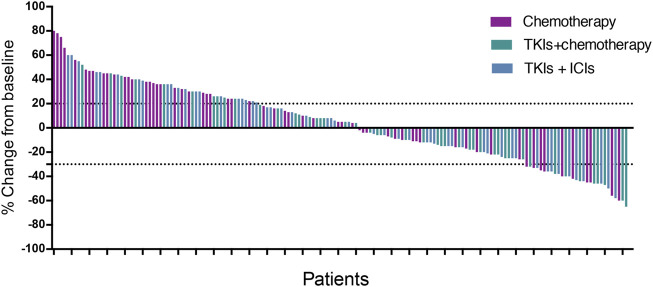
Waterfall plot of the best response change.

In this study, no patient achieved CR after treatment. After treatment, 29 patients obtained PR, 75 patients and 58 patients had SD and PD, respectively. The ORR and DCR in the present study were 17.9% (29/162) and 64.2% (104/162), respectively (Table 3). In the chemotherapy group, after treatment, 10 patients obtained PR, 23 and 28 patients had SD and PD, respectively. The ORR and DCR in the chemotherapy group were 16.4% (10/61) and 54.1% (33/61), respectively. In the TKIs + chemotherapy group, after treatment, 9 patients obtained PR, 28 and 10 patients had SD and PD, respectively. The ORR and DCR in the TKIs + chemotherapy group were 19.1% (9/47) and 78.7% (37/47), respectively. In the TKIs + ICIs group, after treatment, 10 patients obtained PR, 24 and 20 patients had SD and PD, respectively. The ORR and DCR in the TKIs + ICIs group were 18.5% (10/54) and 63.0% (34/54), respectively. The waterfall plot of the best response change after treatment from baseline was shown in Figure 1. There was no statistically significant difference in ORR among groups (16.4% vs. 19.1% vs. 18.5%, *p* = 0.924). Patients who received TKIs plus chemotherapy obtained better DCR compared with the chemotherapy group (78.7% vs. 54.1%, *p* = 0.008). However, no statistically significant difference was found in DCR between the TKIs + ICIs group and the chemotherapy group (63.0% vs. 54.1%, *p* = 0.336).

**TABLE 3:** Efficacy of chemotherapy, TKIs + chemotherapy and TKIs + ICIs in patients with advanced or metastatic gastric cancer.

Parameter	Best response				ORR	*P*	DCR	*P*	Median PFS (95%CI)	*P*	Median OS (95%CI)	*P*
	CR	PR	SD	PD										
Total	0	29	75	58	17.9 (29/162)	—	64.2% (104/162)	—	3.0 (2.7–3.3)	—	6.0 (5.0–7.0)	—
Treatment programs						0.924		0.029		0.010		0.026
Chemotherapy	0	10	23	28	16.4 (10/61)	—	54.1 (33/61)	—	2.8 (2.1–3.5)	—	5.8 (5.5–6.1)	—
TKIs + chemotherapy	0	9	28	10	19.1 (9/47)	0.709^a^	78.7 (37/47)	0.008^a^	3.3 (2.3–4.3)	0.001^a^	8.0 (4.0–12.0)	0.005^a^
TKIs + ICIs	0	10	24	20	18.5 (10/54)	0.764^a^	63.0 (34/54)	0.336^a^	3.0 (2.4–3.6)	0.051	5.2 (3.4–7.0)	0.260
Type in TKIs + chem						0.132		0.703		0.129		0.764
Apatinib + chem	0	7	14	5	26.9 (7/26)		80.8 (21/26)		4.2 (2.3–6.1)		8.0 (1.8–14.2)	
Anlotinib + chem	0	2	14	5	9.5 (2/21)		76.2 (16/21)		3.2 (2.9–3.5)		7.5 (3.0–12.0)	
Type in TKIs + ICIs						0.658		0.477		0.062		0.216
Apatinib + ICIs	0	4	13	8	16.0 (4/25)		68.0 (17/25)		2.4 (1.7–3.1)		6.0 (3.2–8.8)	
Anlotinib + ICIs	0	6	11	12	20.7 (6/29)		58.6 (17/29)		3.0 (0.4–5.6)		5.0 (2.4–7.6)	

Abbreviations: CR, complete response; PR, partial response; SD, stable disease; PD, progressive disease; ORR, overall response rate; DCR, disease control rate; PFS, progression free survival; OS, overall survival; TKI, tyrosine kinase inhibitors; ICIs, immune checkpoint inhibitors.

^a^ compared with chemotherapy group.

**FIGURE 2:**
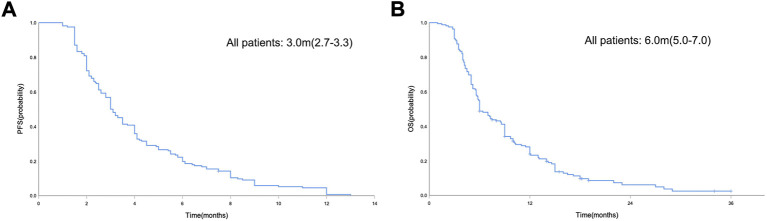
Kaplan-Meier curve of PFS **(A)** and OS **(B)** in the general population.

**FIGURE 3:**
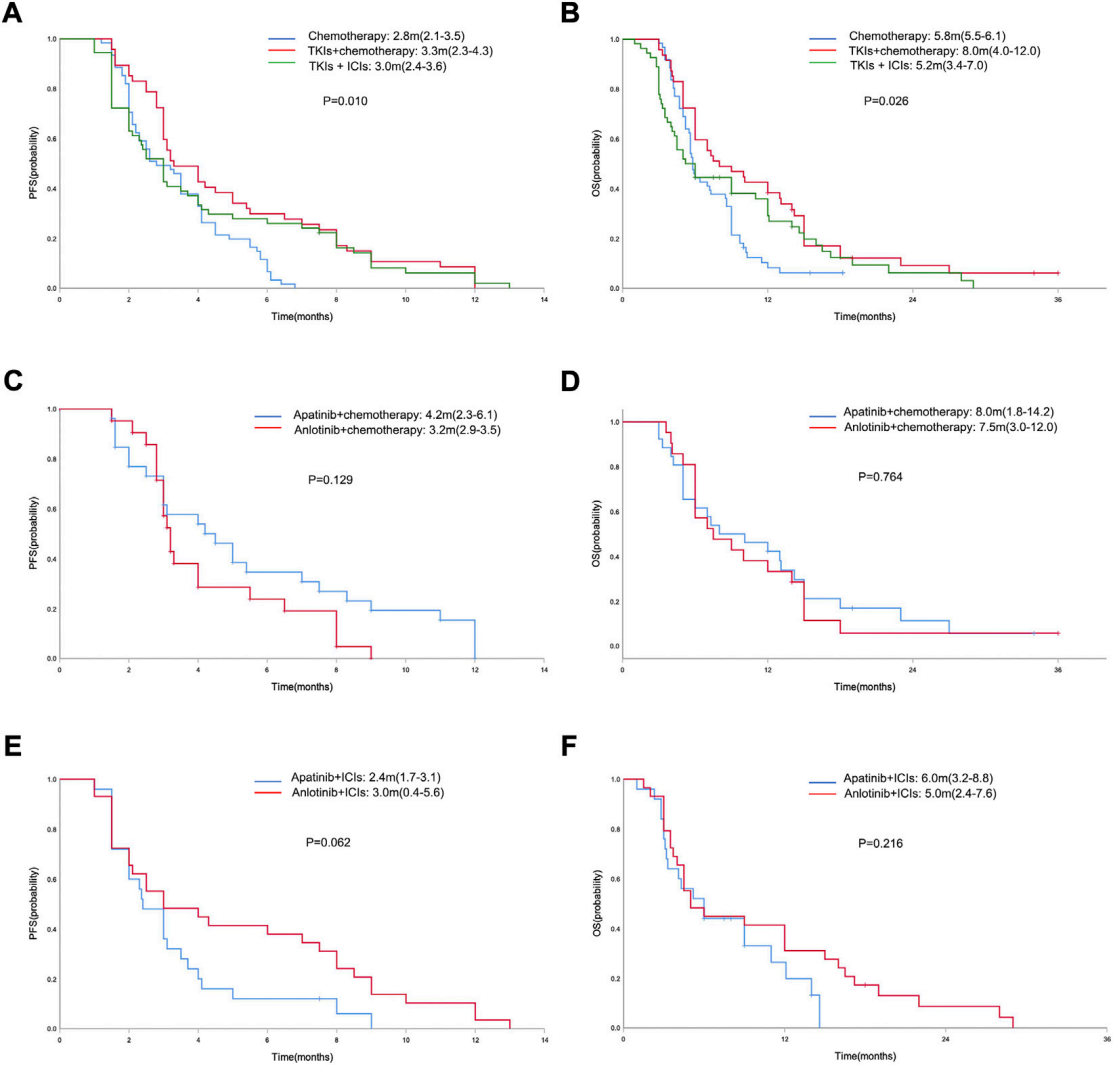
Kaplan-Meier curve of PFS **(A)** and OS **(B)** in the chemotherapy, TKIs + chemotherapy and TKIs + ICIs population. Kaplan-Meier curve of PFS **(C)** and OS **(D)** in the TKIs + chemotherapy group with respect to treatment type (apatinib plus chemotherapy vs. anlotinib plus chemotherapy). Kaplan-Meier curve of PFS **(E)** and OS **(F)** in the TKIs + ICIs group with respect to treatment type (apatinib plus ICIs vs. anlotinib plus ICIs).

**FIGURE 4:**
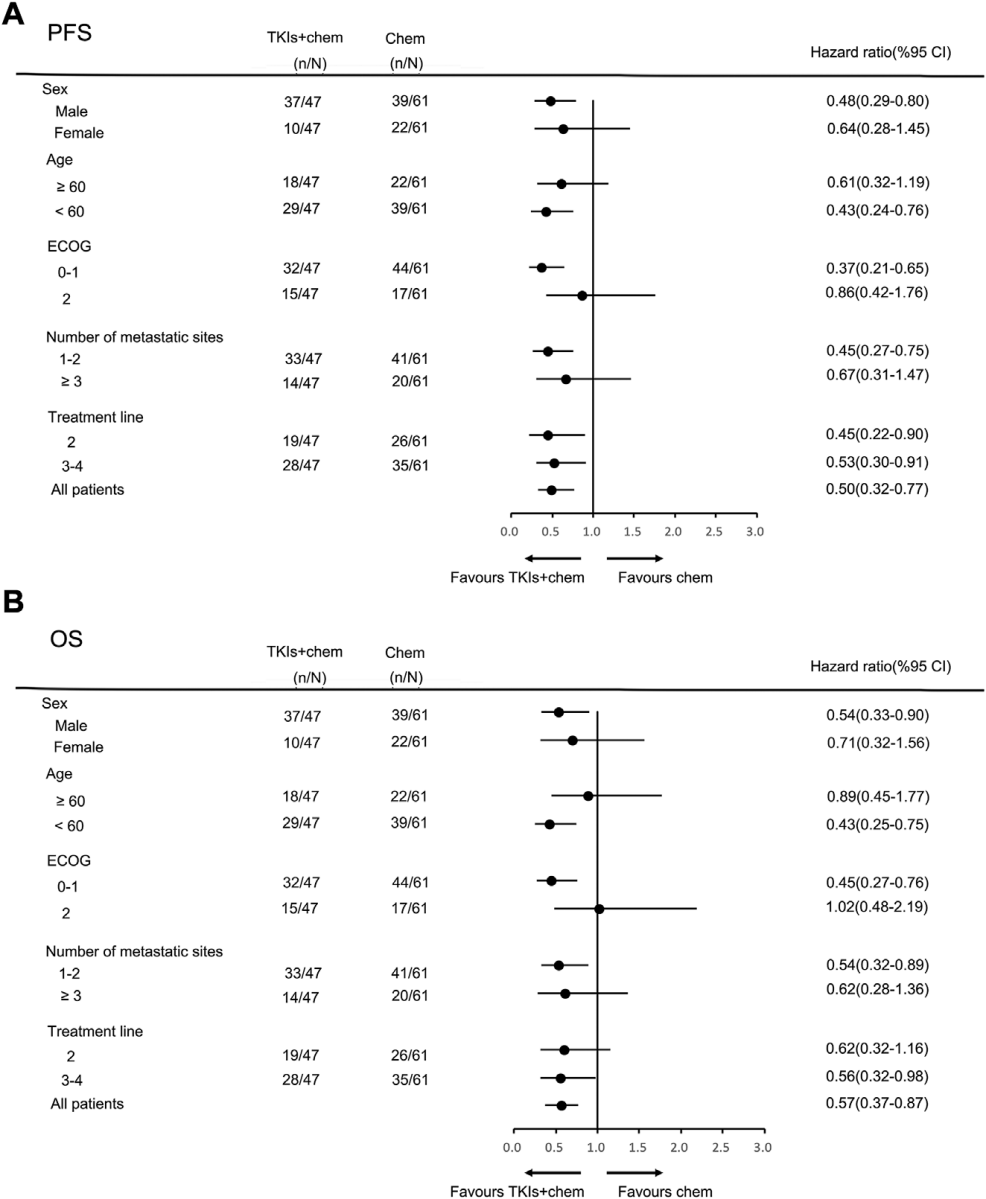
Subgroup analysis of PFS **(A)** and OS **(B)** according to clinicopathologic factors in TKIs + chemotherapy group.

**FIGURE 5:**
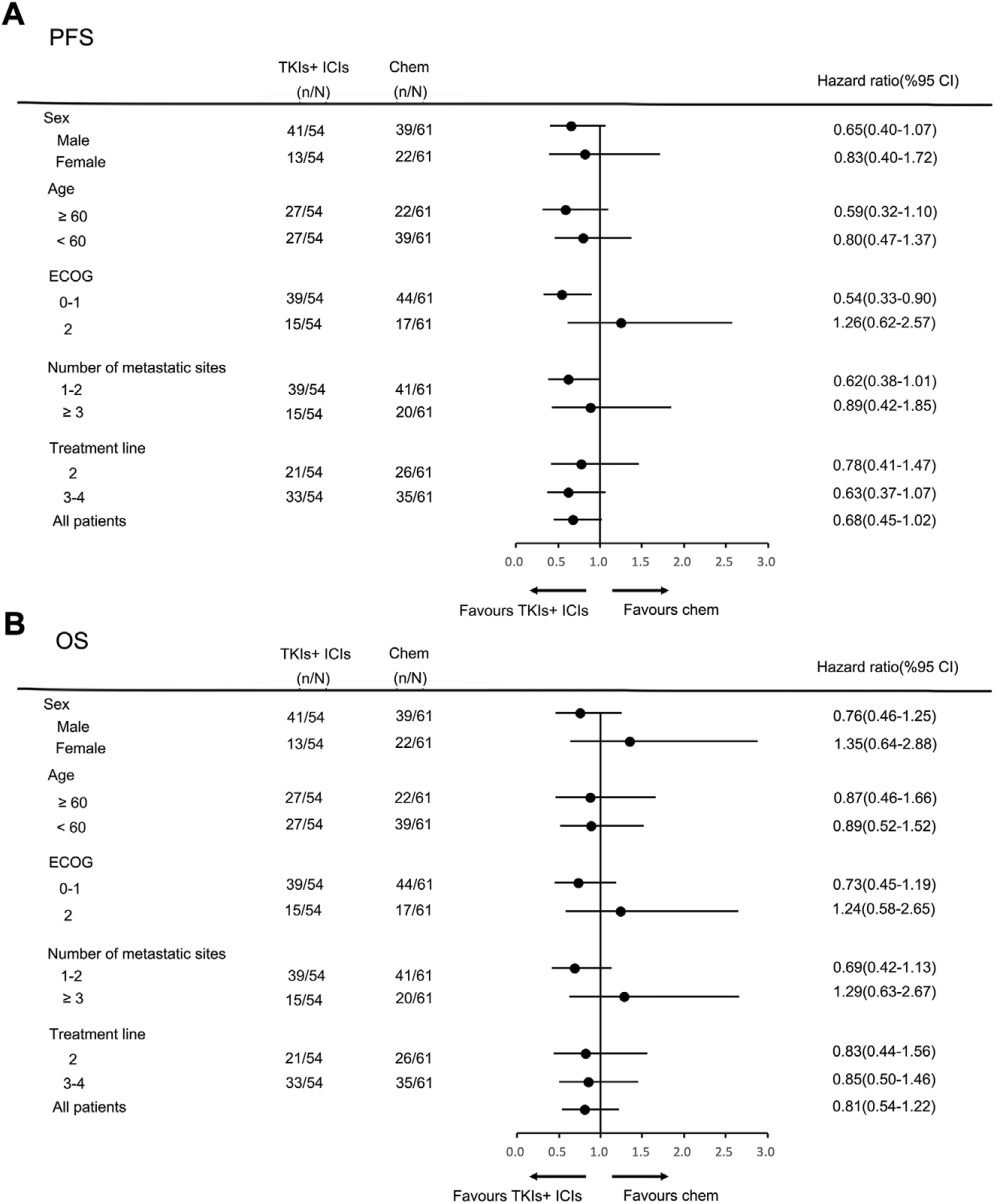
Subgroup analysis of PFS **(A)** and OS **(B)** according to clinicopathologic factors in TKIs + ICIs group.

In the general population, the median PFS and median OS were 3.0 months (95% CI = 2.7–3.3) and 6.0 months (95% CI = 5.0–7.0), respectively (Figures 2A,B). The median PFS in the chemotherapy, TKIs + chemotherapy and TKIs + ICIs population were 2.8 months (95% CI = 2.1–3.5), 3.3 months (95% CI = 2.3–4.3) and 3.0 months (95% CI = 2.4–3.6), respectively (*p* = 0.010; Figure 3A). The median OS in the three groups were 5.8 months (95% CI = 5.5–6.1), 8.0 months (95% CI = 4.0–12.0) and 5.2 months (95% CI = 3.4–7.0), respectively (*p* = 0.026; Figure 3B). The median PFS (3.3 m vs. 2.8 m, *p* = 0.001) and OS (8.0 m vs. 5.8 m, *p* = 0.005) in TKIs plus chemotherapy group were superior to the chemotherapy group. Consistent results were observed in subgroup analysis, including sex, age, ECOG, number of metastatic sites and treatment line (Figure 4). However, no significant difference was found in PFS (*p* = 0.051) and OS (*p* = 0.260) between the patients who received TKIs + ICIs therapy and chemotherapy (Figure 5). In the TKIs + chemotherapy group, no statistically significant differences were found in ORR, DCR, PFS and OS concering treatment type (apatinib plus chemotherapy vs. anlotinib plus chemotherapy; Figures 3C,D). Similar results were also found in the TKIs plus ICIs population (Figures 3E,F).

### Safety

Safety data of the chemotherapy, TKIs + chemotherapy and TKIs + ICIs groups were consistent with the known safety profiles of relevant drugs (Table 4). No unexpected side effects or treatment-related death were observed. Most of the adverse events were grade 1–2 in severity and the incidence of Grade 3–4 AEs were 31.1%, 38.3% and 18.5%, respectively. Different treatment manners present with special spectrum of adverse events. The incidence of hematological toxicity was higher in the chemotherapy and TKIs + chemotherapy group. Grade 3–4 hematological AEs in chemotherapy and TKIs + chemotherapy group were decreased white blood count (n = 6, 9.8%; n = 5, 10.6%), decreased neutrophil count (n = 5, 8.2%; n = 5, 10.6%), anemia (n = 2, 3.3%; n = 1, 2.1%) and increased ALT/AST (n = 1, 1.6%; n = 1, 2.1%). In TKIs + chemotherapy and TKIs + ICIs groups, the most common apatinib or anlotinib related non-hematological AEs were secondary hypertension, hand-foot syndrome and proteinuria. Grade 3–4 secondary hypertension occurred in 1 (2.1%) and 5 (9.3%) patients, and grade 3–4 hand-foot syndrome occurred in 3 (6.4%) and 3 (5.6%) patients, respectively. 4 patients in TKIs + chemotherapy and 3 patients in TKIs + ICIs group had gum bleeding, but no severe bleeding events occurred. The most common ICIs -related adverse events in TKIs + ICIs group were rash, pneumonitis and hypothyroidism. Grade 3–4 rash and pneumonitis occurred in 1 (1.9%) and 1 (1.9%) patient, respectively.

**TABLE 4:** Treatment-related AEs (TRAEs).

Adverse event	Chemotherapy		TKIs + chemotherapy		TKIs + ICIs	
	All grade	≥ Grade3	All grade	≥ Grade3	All grade	≥ Grade3
Hematologic						
Decreased white blood count	38 (62.3)	6 (9.8)	29 (61.7)	5 (10.6)	3 (5.6)	0
Decreased neutrophil count	38 (62.3)	5 (8.2)	29 (61.7)	5 (10.6)	3 (5.6)	0
Anemia	18 (29.5)	2 (3.3)	16 (34.0)	1 (2.1)	8 (14.8)	0
Decreased platelet	8 (13.1)	0	6 (12.8)	0	6 (11.1)	0
Increased ALT/AST	10 (16.4)	1 (1.6)	9 (19.1)	1 (2.1)	5 (9.3)	0
Hypercholesterolemia	4 (6.6)	0	4 (8.5)	0	5 (9.3)	0
Hypertriglyceridemia	4 (6.6)	0	3 (6.4)	0	8 (14.8)	0
LDL elevation	2 (3.3)	0	3 (6.4)	0	6 (11.1)	0
TSH elevation	0	0	0 ()	0	11 (20.4)	0
Non-hematologic						
Fatigue	16 (26.2)	0	17 (36.2)	0	20 (37.0)	0
Nausea or Vomiting	20 (32.8)	2 (3.3)	14 (29.8)	1 (2.1)	2 (3.7)	0
Diarrhea	11 (18.0)	1 (1.6)	8 (17.0)	0	2 (3.7)	0
Muscle pain/joint pain	18 (29.5)	0	8 (17.0)	1 (2.1)	0	0
Sensory neurotoxicity	20 (32.8)	2 (3.3)	10 (21.3)	0	0	0
Secondary hypertension	0	0	13 (27.7)	1 (2.1)	20 (37.0)	5 (9.3)
Hand-foot syndrome	0	0	12 (25.5)	3 (6.4)	16 (29.6)	3 (5.6)
Proteinuria	0	0	10 (21.3)	0	13 (24.1)	0
Rash	2 (3.3)	0	6 (12.8)	0	14 (25.9)	1 (1.9)
Pneumonitis	0	0	0 ()	0	5 (9.3)	1 (1.9)
Oral mucositis	4 (6.6)	0	3 (6.4)	0	6 (11.1)	0
Hypothyroidism	0	0	0 ()	0	11 (20.4)	0
Gum bleeding	0	0	4 (8.5)	0	3 (5.6)	0

AE, adverse event.

## Discussion

The cytotoxic chemotherapy drugs represented by irinotecan and taxanes are still the cornerstone of second-line treatment for metastatic gastric cancer, but these traditional chemotherapy drugs have entered a bottleneck period. The randomized and phase III WJOG 4007 clinical study compared the efficacy of irinotecan and paclitaxel in patients with advanced or metastatic gastric cancer after failure of prior fluoropyrimidine plus platinum chemotherapy. The median PFS of paclitaxel and irinotecan was only 3.6 months and 2.3 months (Hironaka et al., 2013). Therefore, It is necessary to find new therapeutic targets and corresponding treatment methods to further improve the prognosis of patients.

In the era of precision medicine, targeted therapy and immunotherapy have achieved promising clinical efficacy. Based on the results of phase 3 ToGA study, trastuzumab combined with chemotherapy has become the standard treatment option for human epidermal growth factor receptor-2 (HER2) positive metastatic gastric cancer (Bang et al., 2010). However, the positive rate of HER-2 in Chinese patients is only 12–13%, and simultaneously the value of continuous use of trastuzumab beyond progression (TBP) as second-line therapy is still controversial (Al-Shamsi et al., 2016; Ter Veer et al., 2018). Until now, the approved anti-angiogenic drugs for metastatic gastric cancer include ramucirumab and apatinib. Phase III RAINBOW study showed that the anti vascular endothelial growth factor receptor 2 (VEGFR2)_monoclonal antibody ramucirumab in combination with chemotherapy significantly improved the OS compared with single-agent paclitaxel chemotherapy (9.63 vs. 7.36 months) (Wilke et al., 2014). However, ramucirumab has not yet been marketed in China. Another phase III clinical study evaluated the efficacy of anti VEGFR-2 small molecule tyrosine kinase inhibitor apatinib, which demonstrated that apatinib significantly improved the median PFS of patients (2.6 vs. 1.8 months) (Li et al., 2016). The prospective ATTRACTION-02 and KEYNOTE-059 studies confirmed the efficacy of immunotherapy as third-line treatment option for metastatic gastric cancer. Table 5 summarized the clinical second-line or above therapy research data for metastatic gastric cancer (Kang et al., 2017). Overall, the current efficacy of traditional chemotherapy drugs has reached a plateau. In the second-line of metastatic gastric cancer, the median PFS of chemotherapy was 2.3–3.6 months. Compared with chemotherapy, anti-angiogenic targeted therapy and immunotherapy provide new strategies for the late-line treatment of metastatic gastric cancer. However, there is still a huge unmet clinical demand.

**TABLE 5:** Summary of clinical research data on second-line or above therapy for advanced or metastatic gastric cancer.

Study	Patients	Cohort	Treatment line	ORR	DCR	Median PFS	Median OS
WJOG 4007	Metastatic or recurrent gastric adenocarcinoma	Paclitaxel	Second-line	—	—	3.6 (3.3–3.8)	9.5 (8.4–10.7)
WJOG 4007	Metastatic or recurrent gastric adenocarcinoma	Irinotecan	Second-line	—	—	2.3 (2.2–3.1)	8.4 (7.6–9.8)
Eliza Hawkes,2011	Oesophagogastric cancer	Docetaxel + irinotecan	Second-line	24.4%	41.5%	11 weeks (9–13)	24 weeks (12–35)
RAINBOW	Advanced gastric or GEJ	Ramucirumab plus paclitaxel vs. placebo plus paclitaxel	Second-line	27.9 vs. 16.1%	80.0 vs. 63.6%	4.4 (4.2–5.3) vs. 2.9 (2.8–3.0)	9.6 (8.5–10.8) vs. 7.4 (6.3–8.4)
RAINBOW-Asia	Metastatic or locally advanced gastric or GEJ	Ramucirumab plus paclitaxel vs. placebo plus paclitaxel	Second-line	26.5 vs. 20.5%	76.8 vs. 71.2%	4.14 (3.71–4.30) vs. 3.15 (2.83–4.14)	8.71 (7.98–9.49) vs. 7.92 (6.31–9.10)
Jin Li,2016	Advanced gastric or GEJ	Apatinib vs. placebo	Third or above	2.84% vs. 0	42.05% vs. 8.79%	2.6 (2.0–2.9) vs. 1.8 (1.4–1.9)	6.5 (4.8–7.6) vs. 4.7 (3.6–5.4)
ATTRACTION-02	Advanced or recurrent gastric or GEJ	Nivolumab or placebo	Third or above	11.2% vs. 0	40.3% vs. 25%	1.61 (1.54–2.30) vs. 1.45 (1.45–1.54)	5.26 (4.60–6.37) vs. 4.14 (3.42–4.86)
KEYNOTE-059	Metastatic gastric or GEJ	Pembrolizumab	Third or above	11.6%	27.0%	2.0 (2.0–2.1)	5.6 (4.3–6.9)

Some recent studies suggest that anti-angiogenic targeted TKIs combined with chemotherapy or immunotherapy have shown a good coordination effect and have become a new exploration strategy. Our present study enrolled 162 patients with advanced or metastatic gastric cancer and evaluating the efficacy and safety of chemotherapy, TKIs + chemotherapy and TKIs + ICIs. To our knowledge, this is the first and largest real-world study comparing clinical efficacy of traditional chemotherapy and novel combination therapy strategy. Although no significant difference was found in ORR between chemotherapy and TKIs + chemotherapy, patients who received TKIs plus chemotherapy obtained better DCR (78.7% vs. 54.1%) and superior survival. Median PFS of 3.3 months and median OS of 8.0 months were obtained in TKIs plus chemotherapy group. In RAINBOW-Asia study, which also evaluated the efficacy of anti-angiogenic ramucirumab combined with chemotherapy, the DCR was 76.8%, median PFS and OS were 4.14 and 8.71 months, respectively (Xu et al., 2021). Unlike the RAINBOW-Asia study, which enrolled all patients receiving second-line therapy, the proportion of patients who received TKIs plus chemotherapy as second-line therapy in our present study was only 40.4%, the other 59.6% patients as third to fourth-line treatment. Unlike monoclonal antibody anti-angiogenic drugs, small-molecule multi-targeted TKIs can target multiple receptor sites simultaneously, including VEGFR, c-Kit, c-Met, fibroblast growth factor receptor (FGFR) and platelet-derived growth factor receptor (PDGFR). However, monotherapy with TKIs has shown limited efficacy in most cancers. We reviewed relevant literature, and there are few basic studies on the mechanism of action of anti-angiogenic TKIs combined with chemotherapy. Preclinical studies have shown that anti-angiogenic agents can normalize blood vessels in tumor tissue, thus sustaining the therapeutic efficacy of chemotherapy (Lennernas et al., 2003; Wang et al., 2021a). Several recent studies have confirmed the efficacy of TKIs plus chemotherapy in non-small cell lung cancer (NSCLC), ovarian cancer, and etc., (Bondarenko et al., 2015; Lan et al., 2018; Nie et al., 2021). Our present study confirmed the value of anti-angiogenic TKIs plus chemotherapy for metastatic gastric cancer. We will continue to pay attention to reports of basic research and clinical trails related to anti-angiogenic TKIs combined with chemotherapy in metastatic gastric cancer.

Anlotinib was not approved for the treatment of gastric cancer. There are two reasons for why we use this agent. First, all the 162 patients had undergone previous systematic therapy, 40.7% received chemotherapy, TKIs + chemotherapy or TKIs + ICIs therapy as second-line therapy, and as third-fourth line therapy in the other 59.3% patients. Therefore, some of the patients who received anlotinib in this study were patients who failed from apatinib treatment. Second, a small number of patients developed intolerable toxicity after receiving apatinib, so they were switched to anlotinib treatment. Although anlotinib was not approved, several retrospective and real-world studies have confirmed the efficacy of anlotinib in metastatic gastric cancer. And simultaneously, before the treatment, we conducted adequate communication and informed notification.

The phase Ib REGONIVO study evaluated the antitumor activity of nivolumab plus regorafenib in the gastric and colorectal cancer cohort. The median PFS of gastric and colorectal cancer patients was 7.9 and 5.6 months, and the 12-month PFS rate reached 41.8% and 22.4%, respectively (Fukuoka et al., 2020). It was considered that anti-angiogenesis therapy was a potential method to reverse immunotherapy resistance. Some clinical studies also explored the efficacy of bevacizumab combined with a PD-1 inhibitor, but the results could not confirm the value of bevacizumab combined with immunotherapy (Moore et al., 2021). This study suggests for the first time that TKIs plus ICIs may become a novel and effective treatment strategy for gastrointestinal tumors. Basic research has shown that anti-angiogenic TKIs can relieve the immunosuppression caused by Treg and TAM cells on T cells by inhibiting colony-stimulating factor 1 receptor (CSF1R) and VEGFR to enhance the efficacy of immunotherapy (Bai et al., 2021; Doleschel et al., 2021). Several previous studies have confirmed the value of anti-angiogenic TKIs plus immunotherapy in renal cell carcinoma, colorectal cancer, NSCLC etc (Atkins et al., 2018; Wang et al., 2021b). However, the efficacy of anti-angiogenic TKIs plus immunotherapy in gastric cancer is still controversial. REGONIVO study results show that the ORR of regorafenib combined with nivolumab in the treatment of gastric cancer is as high as 44%. The LEAP-005 trial showed that the ORR of lenvatinib plus pembrolizumab in patients with previously treated gastric cancer was only 10%. The JVDF trial evaluated the efficacy of ramucirumab plus pembrolizumab in patients with previously treated advanced gastroesophageal cancer, the ORR was only 7% and the median PFS was 2.5 months (Herbst et al., 2019). In our present study, compared with chemotherapy, TKIs plus ICIs as second-line or above therapy did not significantly improve ORR, DCR, median PFS and OS in gastric cancer. And thus, our present study did not support the application of anti-angiogenic TKIs plus ICIs for metastatic gastric cancer.

From previous clinical studies, there is no evidence that the combination of anti-angiogenic TKIs plus chemotherapy or immunotherapy will increase the proportion of adverse events (Wang et al., 2020; Laccetti et al., 2021). The most common adverse events in the chemotherapy and TKIs + chemotherapy group were hematological toxicity. Adding TKIs on basis of chemotherapy did not increase the risk of all grade and grade 3–4 of decreased white blood or neutrophil count, anemia and decreased platelet. Our present study also had limitations because this was an observational study rather than a randomized controlled clinical study. Thus, future validation clinical trials would be needed to confirm the value of TKIs + chemotherapy and TKIs + ICIs as second-line and beyond therapy for patients with metastatic gastric cancer.

In conclusion, these data confirm that compared with chemotherapy, TKIs plus chemotherapy provide a superior second-line or above therapeutic strategy in patients with advanced or metastatic gastric cancer with well tolerated toxicity. However, TKIs + ICIs failed to demonstrate a clinical advantage over chemotherapy.

## Data Availability

The raw data supporting the conclusion of this article will be made available by the authors, without undue reservation.
